# Preventive correction of fibrinolysis with epsilon aminocaproic acid detected by thromboelastometry during liver transplantation

**DOI:** 10.1590/0102-67202025000022e1891

**Published:** 2025-08-18

**Authors:** José Carlos Rodrigues NASCIMENTO, Luiz Henrique FREITAS, Daniel Vieira PINTO, Antônia Lima SOUZA, Cristhyane Costa AQUINO, Denise Teixeira SANTOS, Rogean Rodrigues NUNES

**Affiliations:** 1 Universidade da Integração Internacional da Lusofonia Afro-Brasileira, Institute of Health Sciences – Redenção (CE), Brazil.; 2 Hospital Geral de Fortaleza, Department of Anesthesia and Liver Transplantation – Fortaleza (CE), Brazil.; 3 Universidade Federal do Ceará, School of Medicine, Department of Morphology and Institute of Biomedicine, Laboratory of the Biology of Tissue Healing, Ontogeny and Nutrition – Fortaleza (CE), Brazil.; 4 Universidade Federal do Amazonas, Hospital Universitário Getulio Vargas, Department of Rehabilitation – Manaus (AM), Brazil.; 5 Rede D’Or, Hospital São Carlos Hospital – Fortaleza (CE), Brazil.

**Keywords:** Liver transplantation, Fibrinolysis, Thromboelastography, Antifibrinolytic agents, Transplante de Fígado, Fibrinólise, Tromboelastografia, Antifibrinolíticos

## Abstract

**Background:**

Orthotopic liver transplantation (OLT) is a highly complex procedure, which can be difficult to control intraoperatively in patients with coagulopathies.

**Aims:**

The aim of this study was to evaluate the prophylactic administration of epsilon aminocaproic acid (EACA) to reduce the need for transfusion of blood products and its relevance for thrombosis.

**Methods:**

Patients were randomized into two groups: one group received EACA (20 mg/kg/h) before surgical incision until the end of OLT and a control group received a similar volume of 0.9% saline solution. Blood was collected to analyze fibrinolysis and coagulation disorders using rotational thromboelastometry (ROTEM®).

**Results:**

A total of 24 patients received EACA and 26 patients received saline solution. In the analysis of the fibrinolytic and hemostatic coagulation profile by ROTEM®, fibrinolysis was significantly less frequent in the group of patients treated with EACA (p<0.001) in the anhepatic phase. There were no significant differences in the other extrinsic pathway thromboelastometry and fibrinogen-specific thromboelastometry analyses. In addition, there were no significant differences between both groups regarding the average and percentage transfusion of blood products, postoperative complications, patients who were discharged from the hospital, and those who died within 3 months after liver transplantation.

**Conclusions:**

Although the administration of EACA did not reduce the transfusion of blood products, this drug effectively treated fibrinolysis and was not associated with any complications with increased risk of vein and hepatic artery thrombosis or mortality within 3 months after liver transplantation.

## ARTICLE HIGHLIGHTS

- Orthotopic liver transplantation (OLT) is a highly complex procedure.
- OLT can be difficult to control intraoperative bleeding in patients with coagulopathies.
- OLT may result in a high need for transfusion of blood products.
- Epsilon aminocaproic acid (EACA) can reduce the need for transfusion of Hood products.
- EACA can be safe with regard to complications such as thrombosis.

## CENTRAL MESSAGE

A total of 105 patients were assessed for eligibility, and 55 were excluded. The remaining 50 patients were randomized, of which 24 patients were allocated to the intervention group and the other 26 to the saline placebo group. In the analysis of the fibrinolytic and hemostatic coagulation profile by rotational thromboelastometry, fibrinolysis was significantly less frequent in patients treated with epsilon aminocaproic acid (p<0.001) compared to those in the placebo group during the anhepatic phase. In the other analyses using thromboelastometry assays such as extrinsic pathway thromboelastometry (EXTEM) (clotting time [CT], clot formation time, alpha angle, amplitude of clot firmness 10 min after CT [A10], and maximum clot firmness [MCF]) and fibrinogen-specific thromboelastometry (FIBTEM) (A10 and MCF), there was no significant difference nor postoperative complications in both groups.

## PERSPECTIVES

Some studies have shown that epsilon aminocaproic acid (EACA) inhibits the binding of plasminogen to lysine residues on the surface of fibrin and prevents conversion of plasminogen to plasmin and the degradation of glycoprotein Ib receptors, thus preserving platelet function. Although EACA did not reduce blood product transfusion, the drug effectively treated all cases and was not associated with any complications of increased risk of hepatic artery and vein thrombosis or mortality within 3 months after orthotopic liver transplantation (OLT). These results support the safety of EACA as the antifibrinolytic drug of choice in OLT. However, future studies involving larger randomized clinical trials and higher doses are needed to further investigate the results.

## VISUAL ABSTRACT

**Figure:**
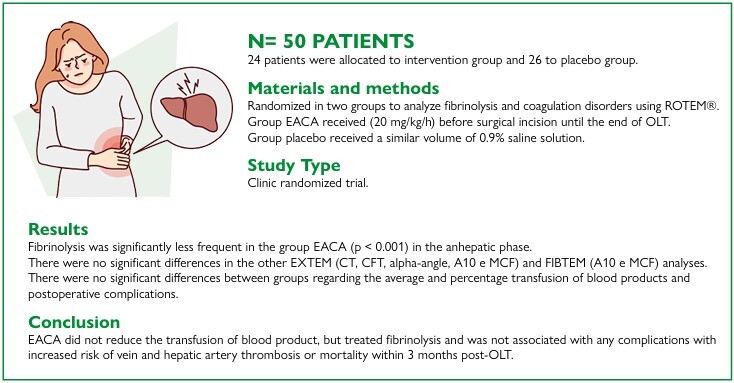

## RESUMO

**Racional:**

O transplante ortotópico de fígado (TOF) é um procedimento altamente complexo, que pode ser de difícil controle intraoperatório em pacientes com coagulopatias.

**Objetivos:**

Avaliar a administração profilática de ácido épsilon aminocapróico (EACA) para reduzir a necessidade de transfusão de hemoderivados e sua relevância para a trombose.

**Métodos:**

Os pacientes foram randomizados em dois grupos: um grupo recebeu EACA (20 mg/kg/h) antes da incisão cirúrgica até o final do TOF, e um grupo controle recebeu um volume semelhante de solução salina a 0,9%. O sangue foi coletado para analisar fibrinólise e distúrbios de coagulação por tromboelastometria rotacional (ROTEM®).

**Resultados:**

Vinte e quatro pacientes receberam EACA e 26 pacientes receberam solução salina. Na análise do perfil de coagulação fibrinolítico e hemostático pelo ROTEM®, a fibrinólise foi significativamente menos frequente no grupo de pacientes tratados com EACA (p<0,001) na fase anepática. Não houve diferenças significativas nas análises de tromboelastometria por outras vias extrínsecas e tromboelastometria específica para fibrinogênio. Além disso, não houve diferenças significativas entre os dois grupos em relação à média e à porcentagem de transfusão de hemoderivados, complicações pós-operatórias, pacientes que receberam alta hospitalar e aqueles que faleceram em até 3 meses após o transplante de fígado.

**Conclusões:**

Embora a administração de EACA não tenha reduzido a transfusão de hemoderivados, este medicamento tratou eficazmente a fibrinólise e não foi associado a quaisquer complicações com aumento do risco de trombose venosa e arterial hepática ou mortalidade em até 3 meses após o transplante de fígado.

## INTRODUCTION

 Orthotopic liver transplantation (OLT) has been established as the only definitive treatment for acute liver failure and endstage chronic liver failure^13^. It is a highly complex procedure and can be difficult to control intraoperative bleeding in patients with coagulopathies, portal hypertension, and/or previous abdominal surgery. Historically, OLT has been associated with excessive intraoperative blood loss and the need to transfuse large amounts of blood products^19^. As a result, the transfusion volume can represent approximately 10% of the transplant costs^2,9^. 

 There are different causes associated with increased blood loss in OLT, such as increased severity of liver disease, poor quality of grafts, low blood reserve, surgical factors, fibrinolysis, portal hypertension, and hemodilution^9,24^. The administration of intraoperative blood products in OLT has been reduced to an average of between 0.5 and 8 units of packed red blood cells per procedure. These results are due to advances in surgical technique, graft preservation, and improvements in anesthetic technique^2,13^. 

 Recent guidelines make few distinctions between surgical techniques and how to better select patients to undergo LSG or RYGB^10,13,20^. 

 The risk of transfusion of allogeneic products expands beyond viral transmission to include allergic reactions, bacterial sepsis, alloimmunization, transfusion-related acute lung injury (TRALI), graft-versus-host disease, volume overload, immunosuppressive effects, and renal failure^1,24^. These risks justify the implementation of bleeding prevention measures such as the administration of synthetic hemostatic products, antifibrinolytic drugs, management of coagulation by rotational thromboelastometry (ROTEM®) (Pentapharm GmbH, Munich, Germany), and recovery of autologous blood by a system cell saver (Medtronic, Minneapolis, MN, USA). Despite these advantages, antifibrinolytics and synthetic products are not exempt from potential complications such as thromboembolism, already documented in some studies^18,21^. 

 Regarding antifibrinolytics, epsilon aminocaproic acid (EACA) combines with plasminogen and free plasmin, preventing the fibrinolytic enzymes from binding to the lysine residues in fibrinogen molecules. Some studies have shown that it inhibits the binding of plasminogen to lysine residues on the surface of fibrin and prevents conversion of plasminogen to plasmin and the degradation of glycoprotein Ib receptors, thus preserving platelet function. EACA is renally eliminated over a 12-h period, and the plasma half-life is relatively short, about 2 h^33^. Following surgery or trauma, hyperfibrinolysis can result in clotting disorders, bleeding, and inflammatory responses. Antifibrinolytic drugs are routinely used to reduce allogeneic blood transfusion, bleeding, and adverse clinical consequences^17^. Tranexamic acid (TXA) and EACA are low-cost and effective agents associated with low mortality compared to aprotinin^18,33^. 

 The ROTEM® is a coagulation-monitoring device that evaluates the viscoelastic properties of blood samples under low-shear conditions. It continuously graphs the firmness of a blood clot during its formation process (coagulation factors and inhibitors, platelets, and fibrin) and the subsequent fibrinolysis. ROTEM® system reagents activate the coagulation system reproducibly, shortening the time to obtain results. This allows for rapid diagnosis and localization of changes in specific pathways of the hemostasis system and analysis of the effect of drugs, thus being the first step toward specific therapy^12,22^. 

 Antifibrinolytics prevent the weakness and deterioration of blood clots by inhibiting plasmin, an enzyme that leads to fibrinolysis. In addition, they contribute to clot stabilization, reducing blood loss and transfusion. However, although previous studies have already evaluated the efficacy and safety of antifibrinolytics in different surgeries, we set out to investigate whether EACA is effective in reducing the need for transfusion of blood products and whether it is safe with regard to complications such as thrombosis. 

## METHODS

 This prospective, randomized, double-blind study was conducted at the Fortaleza General Hospital (FGH) after completing a bibliographic review and gaining approval from the Ethics Committee (CAAE 23142613.6.0000.5040). This study was published in the Brazilian Registry of Clinical Trials (ReBEC) under ensaiosclinicos.gov.br/rg/RBR-105p888f. All patients received information about the study. Informed consent was obtained from each patient. 

 Patients submitted to OLT from May 2017 to July 2021 of both sexes and aged 18 years or older were included. Exclusion criteria were patients with a contraindication to antifibrinolytic therapy, such as disseminated intravascular coagulation (DIC), bacteremia, portal vein and hepatic artery thrombosis, Budd-Chiari syndrome, neoplasia, autoimmune hepatitis, and acute or chronic renal failure and those who refused to participate in the study. 

 Participants were randomized to receive EACA (20 mg/kg/h) in a continuous intravenous infusion before the surgical incision until the end of OLT, and a placebo group received a similar volume of 0.9% saline solution. Randomization was double-blind in a sealed envelope. Neither surgeons nor anesthesiologists were aware of the group to which the patients had been allocated. 

 To prepare the solution, 10 ampoules (1 g/20 mL) of EACA were diluted in 300 mL of 0.9% saline solution, obtaining a 20 mg/mL solution that was administered in a continuous infusion at a dose of 20 mg/kg/h, using a maximum of 10 g intraoperatively. 

 All patients underwent the same Piggyback surgical technique. They also received the same anesthetic technique, hemodynamic monitoring, and coagulation monitoring using ROTEM®. In addition, all received immunosuppression in the anhepatic phase (AP) with 500 mg of methylprednisolone. The same surgical team performed all procedures during the study period. 

 To maintain hemodynamics, 2% albumin solutions were prepared (500 mL of Ringer’s lactate solution+50 mL of 20% human albumin). Additionally, noradrenaline (0.05 μg/kg/min), metaraminol, atropine, and adrenaline were prepared for use in situations of hemodynamic instability. The ventilatory parameters were a tidal volume of 6–8 mL/kg ideal body weight, a fraction of inspired oxygen of 40% in the mixture with medical air, and an end-expiratory pressure of 5 cm of H_2_O. 

 During the surgical procedure, blood samples were collected hourly for blood gas analysis and three times for thromboelastometry (at the beginning of the surgery [BS] before the skin incision, at the beginning of the vena cava anastomosis in the AP, and at the beginning of the anastomosis of the bile ducts in the neohepatic phase [NP]). When intervention was required, additional blood samples were collected 10 min after each intervention. 

 Blood collection for the measurement of thromboelastometric samples was performed through an arterial catheter with 10 mL disposable syringes using the two-syringe method without heparin, that is, the first 10 mL sample of blood collected was discarded to avoid hemodilution and the second sample after this procedure was used for thromboelastometry analysis. 

 Blood was collected for the analysis of fibrinolysis and coagulation disorders using thromboelastometry assays such as extrinsic pathway thromboelastometry (EXTEM) with the following parameters: clotting time (CT), clot formation time (CFT), alpha angle and maximum clot firmness (MCF), maximum lysis (ML) at 60 min, and fibrinogen-specific thromboelastometry (FIBTEM) with the following parameters: amplitude of clot firmness 10 min after CT (A10) and MCF. Each sample was processed for 60 min for the analysis of fibrinolysis by ML. 

 During surgery, sodium bicarbonate and calcium were administered to maintain the pH=7.3 and ionized calcium levels=1.1 mmol/L. In addition, red blood cell transfusion is indicated to maintain the hemoglobin (Hb)=8 g/dL. Another measure adopted was to maintain the patients’ temperature=35.5°C. These measures are important, as acidosis decreases fibrin synthesis and increases clot lysis, and hypothermia decreases fibrin and clotting factor synthesis and impairs platelet function^8^. 

 Although experiments have shown that the impact of temperature on ROTEM® is minimal for most parameters, the sample should preferably be at a temperature close to 37°C. Therefore, to obtain reproducible and accurate results, mainly for CT and CFT, all samples were submitted to the preheating station in ROTEM® for 5 min before the beginning of the test processing. 

 Once the results of the ROTEM® analysis were obtained, the values were compared with the normal values. If corrections were necessary, along with a clinical diagnosis of hypocoagulation and/or fibrinolysis and microvascular bleeding, they were performed according to the hemostasis protocol (Table 1). 

**Table 1:** Demographic and surgical characteristics of patients undergoing orthotopic liver transplantation.

Variables		EACA (n=24)	Control (n=26)	p-value
Age (years)		54.8±11.6	51.8±11.6	0.285
Weight (kg)		69.4±14.6	71.9±16.3	0.426
MELD (Model for End‑Stage Liver Disease)		22.2±6.8	23.8±6.7	0.726
Gender				
	Male, n (%)	19 (79.2)	18 (69.2)	0.526
	Female, n (%)	5 (20.8)	8 (30.8)	
Causes of liver disease, n (%)				
	Alcoholic	11 (45.8)	12 (46.2)	0.614
	Cryptogenic	5 (20.8)	6 (23.2)	
	Schistosomiasis	3 (12.6)	0 (0.0)	
	Hepatitis C	2 (8.3)	3 (11.5)	
	Autoimmune	2 (8.3)	3 (11.5)	
	Non‑alcoholic steatohepatitis	1 (4.2)	1 (3.8)	
	Retransplant primary graft dysfunction	0 (0.0)	1 (3.8)	
Surgical time (min)	316.5±48.7	334.2±72.6	0.330

Fisher’s exact or Mann‑Whitney test was used.

EACA: epsilon aminocaproic acid; p<0.05: statistically significant.

 Data regarding reoperation and transfusion of blood products within 24 h in the immediate postoperative period were analyzed in both groups. Also, the length of stay in the intensive care unit (ICU) and complications such as pulmonary infection, acute rejection, portal vein and hepatic artery thrombosis, sepsis, acute renal failure, and mortality were investigated in both groups in the immediate postoperative period and until hospital discharge or death. 

 For normality, the D’Agostino, Pearson, and ShapiroWilk tests were performed. We used Fisher’s or Mann-Whitney exact test to compare frequencies or means, respectively. The one-way or two-way analysis of variance (ANOVA) test, followed by the Bonferroni or Kruskal-Wallis and Dunn’s test, was used for multiple comparisons when necessary. A p<0.05 was considered statistically significant. Statistical analysis was performed by the GraphPad Prism 5 (GraphPad Software, Inc., La Jolla, CA, USA). 

 Using program OpenEpi, version 3, we calculated for a power of 80% and an error of 0.05 that a sample size of 50 patients was needed to detect a mean difference of 20% in ML in the AP using a two-sided t-test. 

## RESULTS

**Figure 1:**
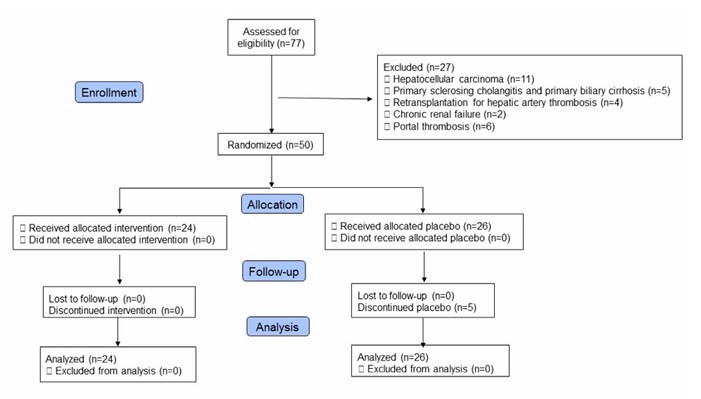
CONSORT diagram.

 We assessed 105 patients for eligibility, and 55 were excluded. The patients were excluded for having hepatocellular carcinoma, primary sclerosing cholangitis, primary biliary cirrhosis, retransplantation for hepatic artery thrombosis, chronic renal failure, and portal thrombosis. There was no refusal of patients to participate in the study. The remaining 50 patients were randomized, of which 24 patients were allocated to the intervention group and 26 to the placebo group. The CONSORT standardized flow diagram shows the enrollment process (Figure 1). We followed the patients for 3 months for mortality or readmissions through telephone calls and return for outpatient consultation. 

 A total of 24 patients received EACA and 26 patients received saline solution. However, only five (19.2%) patients in the placebo group received EACA at a dose of 50 mg/kg because they had fibrinolysis above 15%. There were no significant differences regarding age, weight, Model for End-Stage Liver Disease (MELD), sex, OLT indication, or operating time between the two groups. The most frequent cause in both groups was alcoholic cirrhosis, followed by cryptogenic cirrhosis (Table 1). 

**Figure 2:**
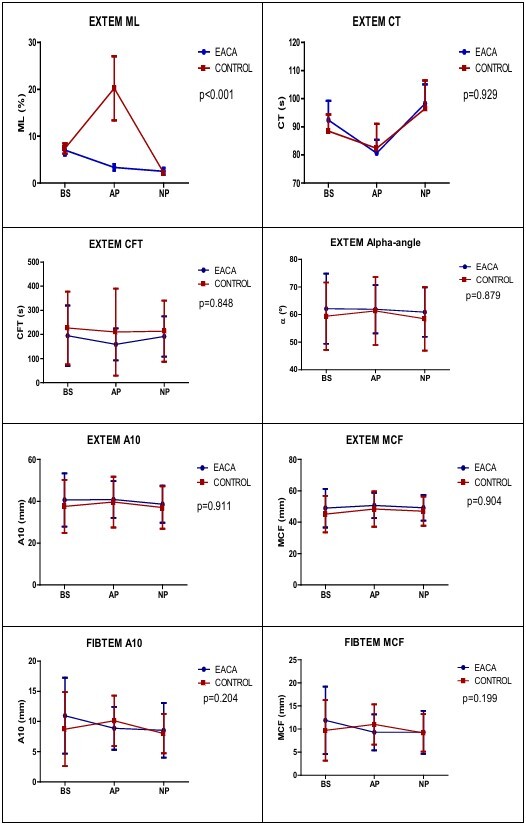
Fibrinolytic and hemostatic profile of coagulation guided by thromboelastometry during orthotopic liver transplantation intraoperative. ML: maximum lysis; EACA: epsilon aminocaproic acid; BS: beginning of surgery; AP: anhepatic phase; NP: neohepatic phase; CT: clotting time; CFT: clot formation time; A10: amplitude of clot firmness 10 min after CT; MCF: maximum clot firmness; p<0.05: statistically significant. Two‑way ANOVA, Bonferroni posttests.

 In the analysis of the fibrinolytic and hemostatic coagulation profile by ROTEM®, fibrinolysis was significantly less frequent in patients treated with EACA (p<0.001) compared to those in the placebo group during the AP. In the other EXTEM (CT, CFT, alpha-angle, A10, and MCF) and FIBTEM (A10 and MCF) analyses, there was no significant difference, as described in Figure 2. 

 Regarding the average transfusion of blood products and/or administration of hemostatic products, there was no significant difference between the two groups, either intraoperatively or within 24 h postoperatively. Also, in the analysis of the percentage of patients who received transfusion of blood products and/or hemostatic products, there was no significant difference between the two groups, both intraoperatively and within 24 h postoperatively (Table 2). 

**Table 2:** Perioperative transfusion support for orthotopic liver transplantation.

Intraoperative	EACA (n=24)	Control (n=26)	p-value
Transfused patients: RBC, n (%)	17 (70.8)	14 (53.8)	0.255
RBC transfused (units, mean±SD)	1.92±2.10	1.62±2.50	0.309
Transfused patients: FFP, n (%)	5 (20.8)	6 (25.0)	1.000
Transfused FFP (units, mean±SD)	0.50±1.10	0.58±1.14	0.840
Patients received: CRYO and/or FC, n (%)	14 (58.3)	19 (73.1)	0.372
Transfused CRYO (units, mean±SD)	5.38±8.71	11.4±12.9	0.072
FC administered (g, mean±SD)	1.96±2.29	1.00±1.77	0.121
Treated patients: PCC, n (%)	11 (45.8)	11 (42.3)	1.000
Administered PCC (IU, mean±SD)	875±1056	885±1143	0.991
Transfused patients: PC, n (%)	7 (29.2)	3 (11.5)	0.164
PC transfused (units, mean±SD)	2.00±3.39	0.69±1.95	0.117
Transfused patients: Cell saver, n (%)	12 (50.0)	15 (57.7)	0.777
Cell saver transfused blood (mL, mean±SD)	311±448	380±480	0.560
Within 24 h after EACA surgery	EACA (n=24)	Control (n=26)	p-value
Transfused patients: RBC, n (%)	10 (41.7)	11 (42.3)	1.000
RBC transfused (units, mean±SD)	2.42±4.39	1.12±2.44	0.573
Transfused patients: FFP, n (%)	5 (20.8)	5 (19.2)	1.000
Transfused FFP (units, mean±SD)	1.79±4.18	0.65±1.72	0.728
Patients received: CRYO and/or FC, n (%)	5 (20.8)	2 (7.69)	0.239
Transfused CRYO (units, mean±SD)	3.87±15.4	0.96±1.37	0.601
FC administered (g, mean±SD)	0.417±1.18	0.00±0.00	NA
Treated patients: PCC, n (%)	1 (4.17)	0 (0.00)	0.480
Administered PCC (IU, mean±SD)	166.7±816.5	0.00±0.00	NA
Transfused patients: PC, n (%)	7 (29.2)	3 (11.5)	0.164
PC transfused (units, mean±SD)	3.25±6.37	0.69±1.95	0.100

Data are presented as number (%) or mean±SD. Mann-Whitney test; Fisher’s exact test.

EACA: epsilon aminocaproic acid; RBC: red blood cells; SD: standard daviation; FFP: fresh‑frozen plasma; CRYO: cryoprecipitate; FC: fibrinogen concentrate; PCC: prothrombin complex concentrate; IU: international unit; PC: platelet concentrate; NA: not evaluated; p<0.05: statistically significant.

 No patient died intraoperatively. There was no statistical difference related to postoperative complications such as pulmonary infection, acute rejection, portal vein and hepatic artery thrombosis, sepsis, reoperation within 24 h of the postoperative period, and acute renal failure in both groups. Also, no significant difference was identified regarding patients discharged from the hospital and those who died within 3 months post-OLT in both groups (Table 3). Only two of the six patients who died in the placebo group had hyperfibrinolysis in the AP. Of the nine patients who died in the postoperative period, six were from the placebo group, three were from the EACA group, two were due to graft rejection, and six were due to a pulmonary infection that progressed to sepsis. 

**Table 3:** Results of variables of patients undergoing orthotopic liver transplantation.

Variables	EACA (n=24)	Control (n=26)	p-value
Lung infection	1 (4.2)	5 (19.2)	0.192
Acute rejection	2 (8.3)	1 (3.8)	0.602
Portal vein and hepaticartery thrombosis	1 (4.2)	2 (7.7)	1.000
Sepsis	2 (8.3)	7 (26.9)	0.142
Reoperation within 24 h after surgery	3 (12.5)	0 (0.0)	0.103
Acute renal failure	3 (12.5)	5 (19.2)	0.704
Hospital discharge	21 (87.5)	20 (76.9)	0.467
Death within 3 months post-OLT	3 (12.5)	6 (23.1)	0.467

Data are presented as numbers (%). Fisher’s exact test.

EACA: epsilon aminocaproic acid; OLT: orthotopic liver transplantation; p<0.05: statistically significant.

## DISCUSSION

 In our study, the ML in EXTEM was significantly higher in the AP of the placebo group. The exclusion of the liver in the AP results in the absence of hepatic synthesis of procoagulant and anticoagulant factors, and non-hepatic clearance of tissue plasminogen activator (t-PA) and circulating activated factors. Thus, as t-PA is the most important endogenous plasminogen activator and as it is still produced by the endothelium, on the other hand, its main antagonist, plasminogen activator inhibitor type 1 (PAI-1), has its production ceased (because it has exclusive hepatic synthesis). This leads to marked hyperfibrinolysis, justifying that the range of the control group patients is very large in the AP than at the BS and in the NP^23,26^. 

 The EACA did not reduce the need for transfusion of blood products, but there was a tendency for the EACA group to receive some more blood components; we speculate that this result may be associated with the monitoring of coagulation by ROTEM® since as soon as fibrinolysis was evidenced in the AP or NP in any group, these patients were treated with EACA at a dose of 50 mg/kg in bolus. This may have strengthened the clot and reduced bleeding in this population. We emphasize that the patients treated with EACA in continuous infusion did not require additional bolus doses of this drug. Also, there was no difference regarding the risk of vein and hepatic artery thrombosis, nor did they die within 3 months post-OLT. 

 The most dangerous complication related to the use of antifibrinolytic drugs is thrombosis^4^. Unlike our findings, administration of aprotinin during OLT was related to thrombotic events^11^. On the other hand, corroborating our study, several studies found that using antifibrinolytic drugs during OLT was safe and not associated with thrombotic complications^3,6,21,32^. 

 Similar to our results, other studies have also found that fibrinolysis was more pronounced in the AP and soon after reperfusion^24,25,28^. Unlike our study, Shimauchi et al.^29^ identified in a retrospective study that fibrinolysis in the pre-hepatic phase was associated with a significant increase in mortality at 30 days and 6 months. 

 A previous study has shown that TXA in high doses reduces blood loss and transfusion^3^. Small doses of TXA reduce fibrinolysis but not transfusion requirements^15^. Aprotinin has also reduced the need for blood transfusion^10,27^. Additionally, TXA, but not EACA, significantly reduces the need for blood transfusion and fibrinolysis^5,6^. In another investigation, TXA was superior in reducing fibrinolysis in the AP compared to aprotinin and placebo^14^. In that study, EACA did reduce fibrinolysis, similar to the results reported by Kong et al.^16^

 In previous investigations comparing TXA and aprotinin or TXA, EACA, and placebo, the investigators found no relevance in complications (thrombosis, reoperation, bleeding, retransplantation, renal failure, and perioperative mortality), corroborating our study^5,7^. 

 Trzebicki et al.^31^ concluded that applying thromboelastometry in intraoperative OLT is an important tool for detecting fibrinolysis and reducing the transfusion of blood products. Stancheva et al.^30^ identified that ROTEM® was preferable and highly informative in OLT hemostatic monitoring. 

 A limitation of this study is that we did not measure bleeding during OLT. Another additional limitation was the small sample size. 

## CONCLUSIONS

 Although the administration of EACA did not reduce the transfusion of blood products, this drug effectively treated hyperfibrinolysis in all cases and was not associated with any complications of increased risk of vein and hepatic artery thrombosis or mortality within 3 months post-OLT. However, there is a need for future larger randomized clinical trials with higher doses of EACA. 

## Data Availability

The information regarding the investigation, methodology, and data analysis of the article is archived under the responsibility of the authors.
